# A Remarkable Case of Epilepsy Caused by Eyestrain

**Published:** 1908-05

**Authors:** George M. Gould

**Affiliations:** Philadelphia, Pa.


### BUFFALO MEDICAL JOURNAL.

VOL. LXIII. MAY, 1908. No. 10

### ORIGINAL COMMUNICATIONS.

### A Remarkable Case of Epilepsy Caused by Eyestrain.

By GEORGE M. GOULD, M.D., Philadelphia, Pa.

ON October 12, 1906, a child of four years of age, to all appearances physically perfect, was sent to me by a general physician of a western city. For a year or two the boy had been having epileptic seizures. No "neurotic" antecedents were to be found in the family history. The father and the mother were as good examples physically of manhood and womanhood as one may find. The father was an educated physician, though not practicing at the present time. No cause for the attacks in the child could be found, except the fancied and "scientific" ones, and they are hardly worth mentioning. Nothing pointed to the eyes. The child was sent to me because I had advocated the truth that in some instances epilepsy is due to eyestrain, and because under the advice and treatment of many of the most famous neurologists, pediatrists, and general physicians of the United States, the child had steadily worsened. After two years of lost and precious time, after two years of injury, after two years of perfect failure, anything, even an eyestrain crank may be tried. The attacks were of all varieties of the two types called *petit mal* and *grand mal.* The total number of all kinds of attacks during the preceding year had been ranging between 437 and 969 per month. The "typical" or *grand mal,* or severest convulsive seizures, with loss of consciousness, etc., numbered from 5 to 25 per month. After one of these attacks it is about twenty minutes before the child can turn his head on the pillow, and the nurse thinks that the attacks in which consciousness is not lost are the worst or more harmful. The child was so stupified and drunk with the bromids which "science" had ordered poured into him for a year or two that a retinoscopic estimate of the refraction was not possible or would have been so inaccurate as to be worthless. There was but one thing to do, that which I had so long and frequently urged upon the profession and yet which I judge had never been done by any practitioner. It will be a century, possibly, before the most valuable and easily carried out method of establishing a differential diagnosis by means of cycloplegia will end doubt, stop useless drugging and mistreatment, in millions of cases of headache and of a hundred kinds of functional disease possibly due to eyestrain. In the case of this poor boy his advisers would, of course, have scouted the idea that his epilepsy could be due to eyestrain, and they would have preferred that he die by bromidism rather than try a harmless drop of atropin to help clear up the etiology of the disease. The greatest pediatric authority in America said the lad had a brain tumor,—*Vale* Pediatrics! The equally great neurologist was equally sure, of course, that "it was a case of pure Jacksonian Epilepsy." The adjective "pure" is comforting, and "Jacksonian" whistles for the trephine. *Vale* Neurology! One leader was mighty sure the disease was "atypical epilepsy." But all diseases are atypical, and "atypical" seems as incurable as "pseudo", or any other disease. The most illuminative diagnosis of another great specialist was pseudo epilepsy, but what to do for the disease, "pseudo," was a question left unanswered. *Vale* pseudology!" More sensible and far more funny was the theory of an attendant who, against the oculist, took the side of scientific medicine. She vigorously protested that not bad eyes, but epilepsy, was the boy's trouble! Of course, the child had been *x*-rayed, stomach contentsed, hematologised, and all that, and all that again. How many thousands of dollars did it all cost? But thanks to a superior wisdom, holes were not bored in the boy's cranium; nor was it sliced like a melon; nor was opportunity given to find "the dura mater adherent," etc., etc. The little less worse was the compelled choice, and for a year or two the boy was made drunk with the diabolic brutal bromid, which never cures and which always curses. Such is the degeneracy of Specialism!

In order to determine if eyestrain was the possible exciting cause of the attacks, I ordered atropinisation of the eyes. But I cared so little for my theory and so much for the patient, that I at once began demanding that the bromid should be stopped. In this I was not successful until a month's time had been lost and a month's injury gained. I was able immediately to secure the consent of the parents that it was better the boy should die of epilepsy than become the lingering idiot of bromidism. Parental love was clear-headed and right-hearted, but "Science" quite naturally preferred the idiocy.

With atropinisation alone the total number of attacks was lessened by about a hundred during the following month. From a written statement of the examining physicians made two weeks after atropinisation of the eyes was begun I quote: "Two things impressed us, his brighter mental condition, and his improved locomotion. There have been no convulsions for eleven days, the longest period of freedom from attacks for three months. He walked better, his mental condition brightened, as shown in conversation, and the like, and he was sleeping better." On November 10, I at last succeeded in getting the bromid stopped. It was somewhat risky perhaps, and I was keenly solicitous of the result. Had the child died I should still have felt I had acted rightly. The child did not die, but something of significant interest occurred. The *petit mal* seizures greatly lessened, in the ensuing month, and the *grand mal* attacks were increased in number. The exact figures are these: out of a total of 404, in November, 59 were of the *grand mal* type.

With the bromid drunkenness removed it was now possible to measure the ametropia by means of the retinoscopic method, and glasses neutralising the boy's compound hyperopic astigmatism were secured. Two noteworthy results followed: The child fought to a victory against the atropin instillations; and he was just as anxious to wear his spectacles. This proved that the atropinisation is of use only temporarily and successful only partially in such cases, and that spectacles are the adequate and proper therapeutic agent. Moreover, it illustrates the old experience that when the right glasses are ordered, and when they are needed by the suffering nervous system, even babies of one, two, or more years of age will welcome them, will not allow them to be removed from the face, and will care for them as well if not better than the grown-ups.

Now comes the startling fact of the history: In the next month, December, after getting the spectacles, although the number of severe convulsive seizures increased somewhat, the total number fell from 404 to 157. In January the total number fell to 85, and in February to 7. Illness in the family during the spring of 1907, made necessary a change of residence for the boy. The journey to another city and the far more irritating environment resulted in a slight increase in the number of seizures. With return to the old home and better conditions, during the summer, six weeks went by without a seizure. At the present time, March, 1908, six months have passed with but two or three slightest *petit mal* hardly noticeable symptoms. The boy is at last natural-minded, and healthy-bodied, "plays with the other children, as a normal child." "His eyes have a bright and normal expression." "He drives the pony, telephones, wears his glasses constantly," etc. He weighs 67½ lbs.

But the illustrious "leaders," the neurologists, and pediatrists, the pseudologists and the atypicalologists, the great ophthalmic surgeons, and diagnosticians all, are today pronouncing upon multitudes of patients the doomful death warrants of "Jacksonian

Epilepsy," "Brain Tumor," "Neurotic Inheritance," "Diathesis," "Autotoxemia," and the rest, and pouring out mystery and bromids with unconcern and self-satisfaction. While official and professorial ophthalmology tries to get its eyebrows in still more highly arched curves as it passes by on the other side. The professor's automobile is in haste on the way to the surgical clinic, or to the learned lecture on organic pathology.

1722 WALNUT STREET.

